# PtWRKY2, a WRKY transcription factor from *Pinellia ternata* confers heat tolerance in *Arabidopsis*

**DOI:** 10.1038/s41598-024-64560-0

**Published:** 2024-06-14

**Authors:** Dan Liu, Wanning Cui, Chen Bo, Ru Wang, Yanfang Zhu, Yongbo Duan, Dexin Wang, Jianping Xue, Tao Xue

**Affiliations:** 1 College of Life Sciences, Huaibei Normal University, Huaibei, 235000 China; 2 College of Agriculture and Engineering, Heze University, Heze, 274015 China; 3 Anhui Provincial Engineering Laboratory for Efficient Utilization of Featured Resource Plants, Huaibei, China; 4 Huaibei Key Laboratory of Efficient Cultivation and Utilization of Resource Plants, Huaibei, China

**Keywords:** WRKY transcriptional factor, High temperature stress, *Arabidopsis*, *Pinellia ternate*, Gene expression, Molecular biology, Plant sciences, Molecular medicine

## Abstract

High temperatures are a major stress factor that limit the growth of *Pinellia ternata*. WRKY proteins widely distribute in plants with the important roles in plant growth and stress responses. However, WRKY genes have not been identified in *P. ternata* thus far*.* In this study, five *PtWRKYs* with four functional subgroups were identified in *P. ternata*. One group III WRKY transcription factor, *PtWRKY2,* was strongly induced by high temperatures, whereas the other four *PtWRKYs* were suppressed. Analysis of transcription factor characteristics revealed that PtWRKY2 localized to the nucleus and specifically bound to W-box elements without transcriptional activation activity. Overexpression of *PtWRKY2* increased the heat tolerance of *Arabidopsis*, as shown by the higher percentage of seed germination and survival rate, and the longer root length of transgenic lines under high temperatures compared to the wild-type. Moreover, *PtWRKY2* overexpression significantly decreased reactive oxygen species accumulation by increasing the catalase, superoxide dismutase, and peroxidase activities. Furthermore, the selected heat shock-associated genes, including five transcription factors (*HSFA1A*, *HSFA7A*, *bZIP28*, *DREB2A,* and *DREB2B*), two heat shock proteins (*HSP70* and *HSP17.4*), and three antioxidant enzymes (*POD34*, *CAT1,* and *SOD1*), were all upregulated in transgenic *Arabidopsis*. The study identifies that *PtWRKY2* functions as a key transcriptional regulator in the heat tolerance of *P. ternata*, which might provide new insights into the genetic improvement of *P. ternata.*

## Introduction

Global warming has led to frequent high-temperature events, and heat stress has become an important issue affecting food security^1,2^. Heat is one of the main abiotic stressors that restrict plant growth and yield formation^3–5^. Research has shown that rising temperatures have significant adverse effects on plant growth, including accelerating plant transpiration, leading to water loss, affecting plant growth and metabolism, reducing plant photosynthetic capacity, and affecting nutrient absorption and growth^5,6^. Plants have developed complex transcriptional regulatory networks to resist high temperatures, and transcription factors play an important role in this network by activating or repressing target genes.

Transcription factor (TF) genes account for a large proportion of the plant genome. For example, there are over 2100 transcription factor genes in *Arabidopsis thaliana* and over 2300 transcription factor genes in rice, which can be divided into the MYB, NAC, AP2/ERF2, and WRKY families based on their protein structure^7^. As one of the largest transcription factor superfamilies, WRKY transcription factors are widely distributed in plants, and all possess the WRKYGQK sequence as well as a C_2_H_2_- or C_2_HC-type zinc-finger motif^8^. Numerous studies have shown that WRKY proteins regulate plant growth and development, aging, secondary metabolism, and environmental responses via specific interactions with the cis-acting element W-box (TTGACC/T) of target genes^9,10^. In terms of high-temperature stress, *AtWRKY39* confers heat tolerance when overexpressed in *Arabidopsis*^11^, and the TaWRKY33 protein can positively regulate high-temperature tolerance in *Arabidopsis*^12^. *AtWRKY25/26/33* have also been shown to positively regulate heat tolerance, and many *WRKY* genes have been found to respond to high temperatures^13^.

*Pinellia ternata* is a herb belonging to the Araceae family that is widely distributed in China and Southeast Asia. Its tubers contain alkaloids, organic acids, and polysaccharides^14,15^, and are used for medicinal purposes. Modern pharmacological studies have shown that *P. ternata* has many medicinal properties, including analgesic, anxiolytic, antitussive, and anticancer effects^16^. The suitable temperature for the growth of *P. ternata* is 15–25 °C. When exposed to high temperatures during growth, *P. ternata* is susceptible to withering, a phenomenon known as “sprout tumble” (ST)^17,18^. ST formation in *P. ternata* shortens its growth period, which is a key limiting factor in tuber production^19^. Therefore, it is important to analyze the ST mechanism and delay the process of ST for improving the yield of *P. ternata* . Thus far, only the functions of *PtSAD* and *PtsHSP* in *P. ternata* in response to high temperatures have been reported^20,21^, and the transcriptional regulatory network of *P. ternata* related to ST is still largely unknown.

In this study, we isolated *WRKY* genes from *P. ternata* based on the full-length transcriptome and identified a *WRKY* gene, *PtWRKY2,* that was significantly induced by high temperatures. Moreover, the function of *PtWRKY2* in high-temperature tolerance was investigated. These data provide new insights into ST mechanisms at the transcriptional level, which could contribute to the genetic improvement of *P. ternata*.

## Results

### Identification and phylogenetic tree analysis of *PtWRKY* genes

Based on the reported transcriptome data for *P. ternata*^19^, five *PtWRKY* genes with serial numbers i1_LQ_Pts1_c14283/f5p11/2027, i1_LQ_Pts1_c61466/f1p0/1914, i1_LQ_Pts1_c39338/f1p8/1965, i2_LQ_Pts1_c2682/f1p6/2136, and i1_HQ_Pts1_c10843/f5p4/1677 were identified and annotated as *PtWRKY1-5*. The predicted coding proteins ranged from 350 amino acids (aa) (PtWRKY2) to 515 aa (PtWRKY1), with molecular masses from 37.79 to 55.07 kD. The isoelectric points varied from 6.1 (PtWRKY5) to 10.15 (PtWRKY2) (Table S2). The five PtWRKYs contained five conserved domains, in which the typical WRKYGQK motif and C_2_HC-type zinc-finger motif appeared, while the C_2_H_2_-type zinc-finger motif appeared in PtWRKY3-5 (Fig. S1).

**Figure 1:**
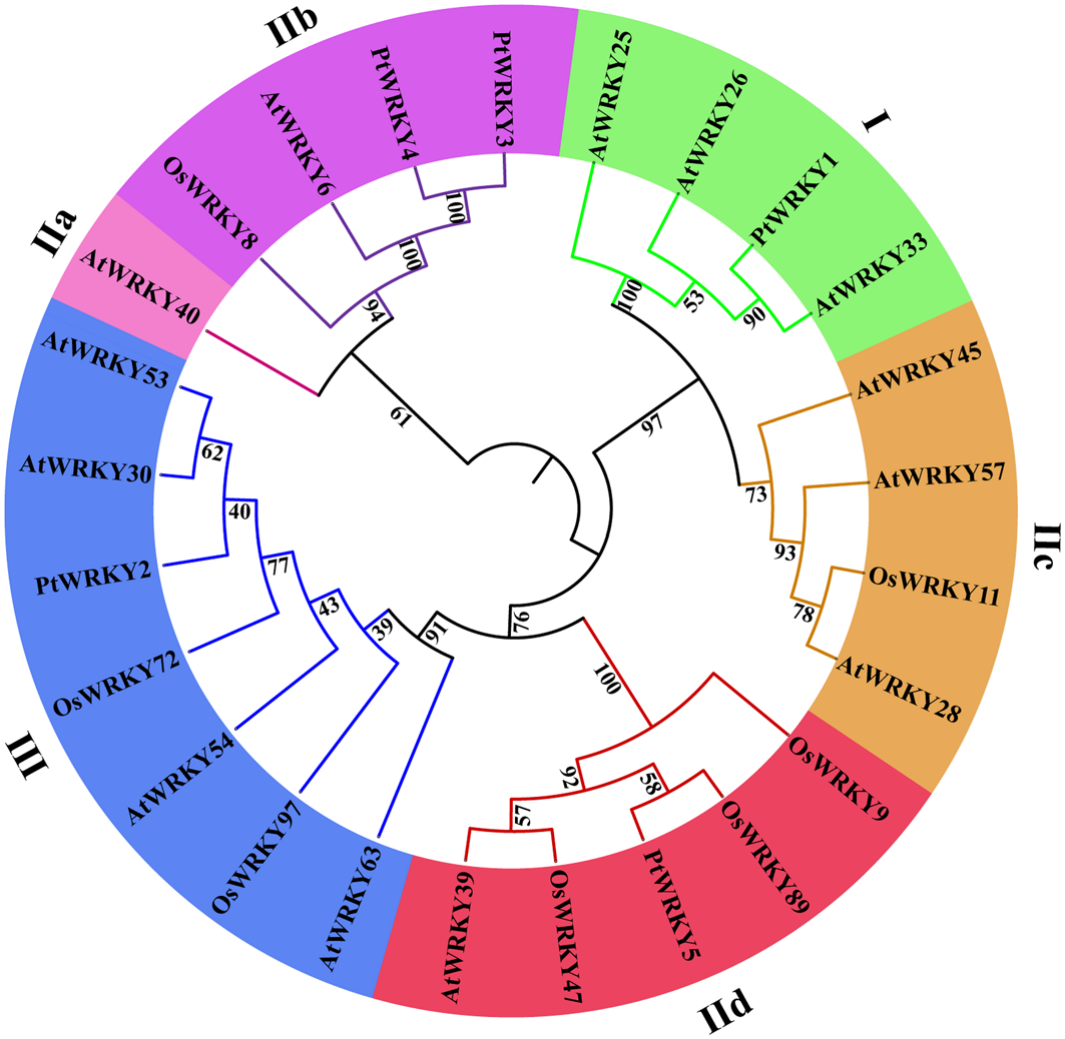
Phylogenetic analysis of the WRKY proteins of *P. ternata*, *Oryza sativa*, and *Arabidopsis* was performed using MEGA7.0, with a bootstrap setting of 1000.

Phylogenetic analysis indicated that the 25 WRKYs from *P. ternata*, *Oryza sativa*, and *Arabidopsis* could be divided into six subclasses. PtWRKY1, AtWRKY25, AtWRKY26, and AtWRKY33 clustered into Group I, whereas PtWRKY2, together with the closely related AtWRKY30, AtWRKY53, and OsWRKY72, were categorized as Group III. PtWRKY3 and PtWRKY4, together with the closest AtWRKY6, belonged to Group IIb and PtWRKY5 clustered with Group IId (Fig. 1).

### Expression pattern analysis of *PtWRKY* genes

**Figure 2:**
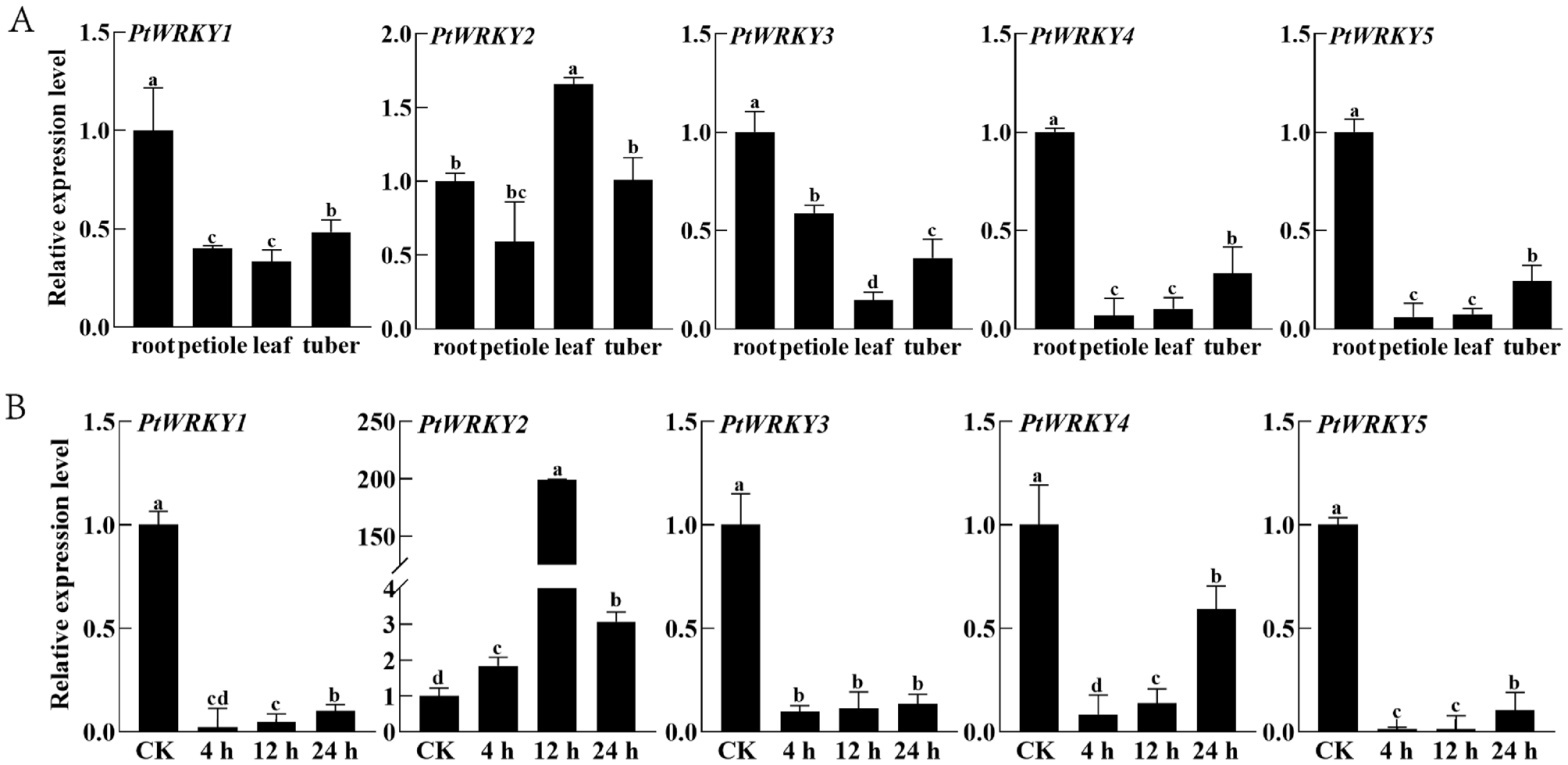
Expression analysis of PtWRKY genes in *P. ternata* using RT-qPCR. (**A**) Expression profiles of PtWRKY genes in the roots, petioles, leaves, and tubers of two-month-old *P. ternata* plants. (**B**) Expression of PtWRKY genes in response to 42 °C heat stress. Values are presented as means ± SD (n = 3). Different letters indicate significant differences at *P* < 0.05.

The expression profiles of the five *PtWRKY* genes were investigated using quantitative real-time PCR. The five *PtWRKY* genes existed in all tissues of *P. ternata*, with *PtWRKY1*, *PtWRKY3*, *PtWRKY4*, and *PtWRKY5* highly expressed in the roots, and *PtWRKY2* highly expressed in the leaves (Fig. 2A). In terms of their heat responses, we observed that only the *PtWRKY2* transcription level significantly increased within 24 h of treatment, with an expression peak at 12 h (nearly 200-fold induction). However, the expression levels of the other four *PtWRKY* genes significantly decreased under stress treatment (Fig. 2B). These results implied that the induction of *PtWRKY2* might participate in the growth regulation of *P. ternata* at high temperatures.

### Transcription factor characteristics of PtWRKY2

**Figure 3:**
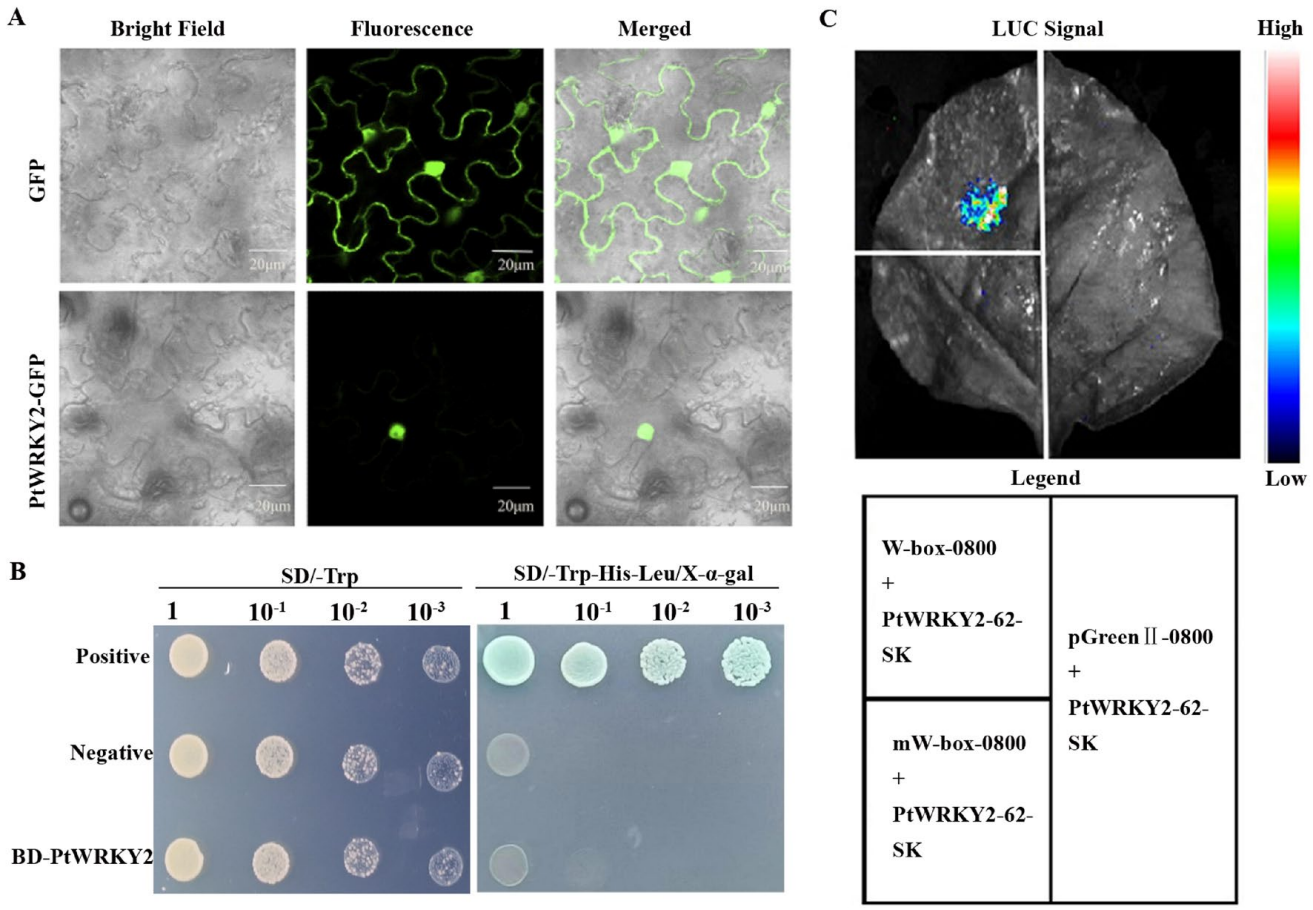
PtWRKY2 characterization. (**A**) Subcellular localization of PtWRKY2 in tobacco leaf epidermal cells. Scale bar = 20 μm. (**B**) Transactivation of PtWRKY2. The construct of pGBKT7-PtWRKY2 was transformed into yeast strain Y2HGold and examined on SD/ − Trp and SD/ − Trp/ − His/ − Ade/X-α-gal plates. (**C**) Dual-LUC assay of PtWRKY2 with three repeats of the W-box (TTGACY) and mW-box (TTTAAY) elements. W-box-0800, mW-box-0800, and pGreenII 0800-LUC empty vectors were transiently expressed in tobacco leaves along with PtWRKY2-62-SK. The LUC signal was captured at 72 h post-transfection.

To identify the biological functions of *PtWRKY2* in response to high temperatures, *PtWRKY2* was selected for further study. First, the PtWRKY2-GFP and GFP vectors were extracted for subcellular localization analysis. The cells were then transformed into tobacco epidermal cells for observation under a fluorescence microscope. As shown in Fig. 3A, the PtWRKY2-GFP fusion proteins were found only in the cell nucleus, whereas the control GFP signals were widely distributed throughout the cell, indicating that PtWRKY2 encodes a nuclear protein.

Second, to confirm the transcriptional activity of PtWRKY2, the plasmids pGBKT7-PtWRKY2, pGBKT7-53 (positive control), and pGBKT7 (negative control) were used for a transcriptional activation assay in Y2HGold yeast cells. The results revealed that all yeast cells transformed with the three vectors grew normally on SD/-Trp plates. Only the positive control grew on the SD/-Trp/-His/-Ade plate, and turned blue with the addition of X-α-gal, whereas the transformants containing pGBKT7-PtWRKY2 and pGBKT7 both failed to grow (Fig. 3B), which suggests that the PtWRKY2 protein did not have transcriptional activation activity.

WRKY transcription factors regulate gene expression by specifically binding to W-box elements within the target gene promoter^22^. To further explore whether PtWRKY2 could bind to W-box, the plasmid PtWRKY2-62-SK was grouped with W-box-0800, mW-box-0800, and pGreenII-0800, respectively. The three combined plasmids were injected into tobacco epidermal cells for the dual-luciferase reporter assay. As shown in Fig. 3C, LUC luminescence signals were produced only when the PtWRKY2-62-SK and W-box-0800 vectors were co-transformed. These results suggest that PtWRKY2 is a typical WRKY protein that specifically binds to W-box elements.

### Overexpression of *PtWRKY2* enhanced the heat tolerance of transgenic *Arabidopsis*

**Figure 4:**
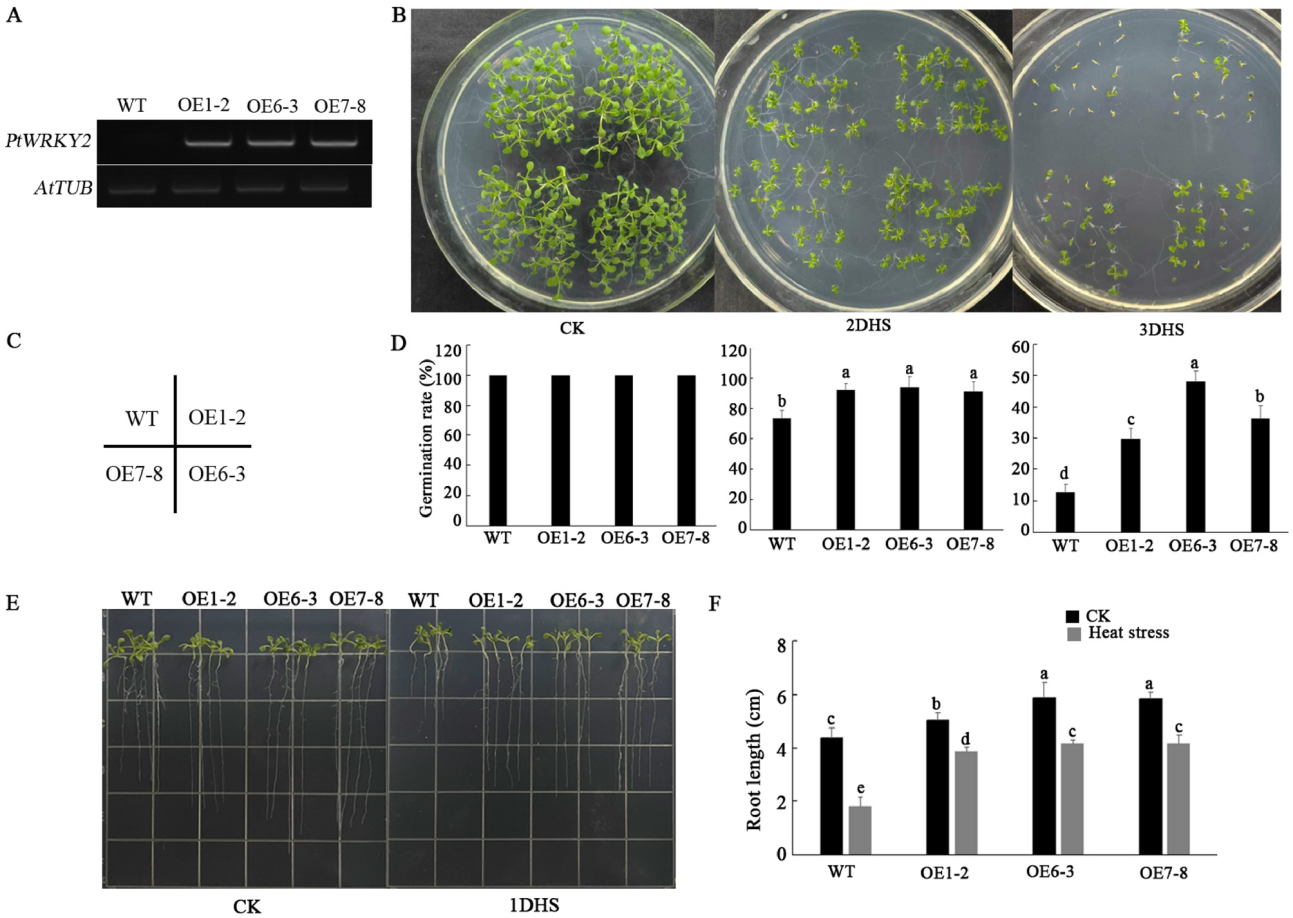
Germination and root elongation analyses of wild-type (WT) and transgenic *Arabidopsis* under 42 °C heat stress. (**A**) *PtWRKY2* expression levels in WT and three T_3_ generation transgenic *Arabidopsis* plants based on RT-PCR. (**B**) Germination of WT and three *PtWRKY2* transgenic lines on 1/2MS medium exposed to 42 °C heat stress for 0, 2, or 3 days. (**C**) Distribution diagram of WT and three *PtWRKY2* transgenic lines on a plate. (**D**) Seed germination rates of WT and transgenic plants within 14 days. Root length (**E**) and statistical analysis (**F**) of WT and three transgenic *Arabidopsis* grown on 1/2MS medium exposed to 42 °C heat stress for 1 day and captured after 10 days. HS represents heat stress. OE1-2, OE6-3, and OE7-8 represent three independent pure transgenic lines. Different letters indicate significant differences at *P* < 0.05.

To confirm the function of *PtWRKY2*, we obtained *PtWRKY2*-overexpressing lines in *Arabidopsis* (OE1-2, OE6-3, and OE7-8) (Fig. 4A), which were used for further analyses in combination with WT *Arabidopsis*. In the seed germination assay, the germination rates of the WT and transgenic lines reached 100% under normal conditions. When exposed to heat stress for two days (2DHS), the germination rate exhibited a downward trend, with a > 90% rate in the transgenic lines and a 73.6% rate in the WT lines. After 3DHS treatment, the germination rate of OE6-3 reached 48.1%, followed by 36.2% in OE7-8, and 31.5% in OE1-2, while only 12.8% of the WT seeds germinated (Fig. 4B–D). The effects of *PtWRKY2* on root length were also evaluated using the above *Arabidopsis* lines. As shown in Fig. 4E,F, the root length of the transgenic lines was greater than that of the WT plants under normal conditions, and root growth was inhibited under high temperature conditions. Notably, root length inhibition was less in the transgenic lines than in the WT plants.

**Figure 5:**
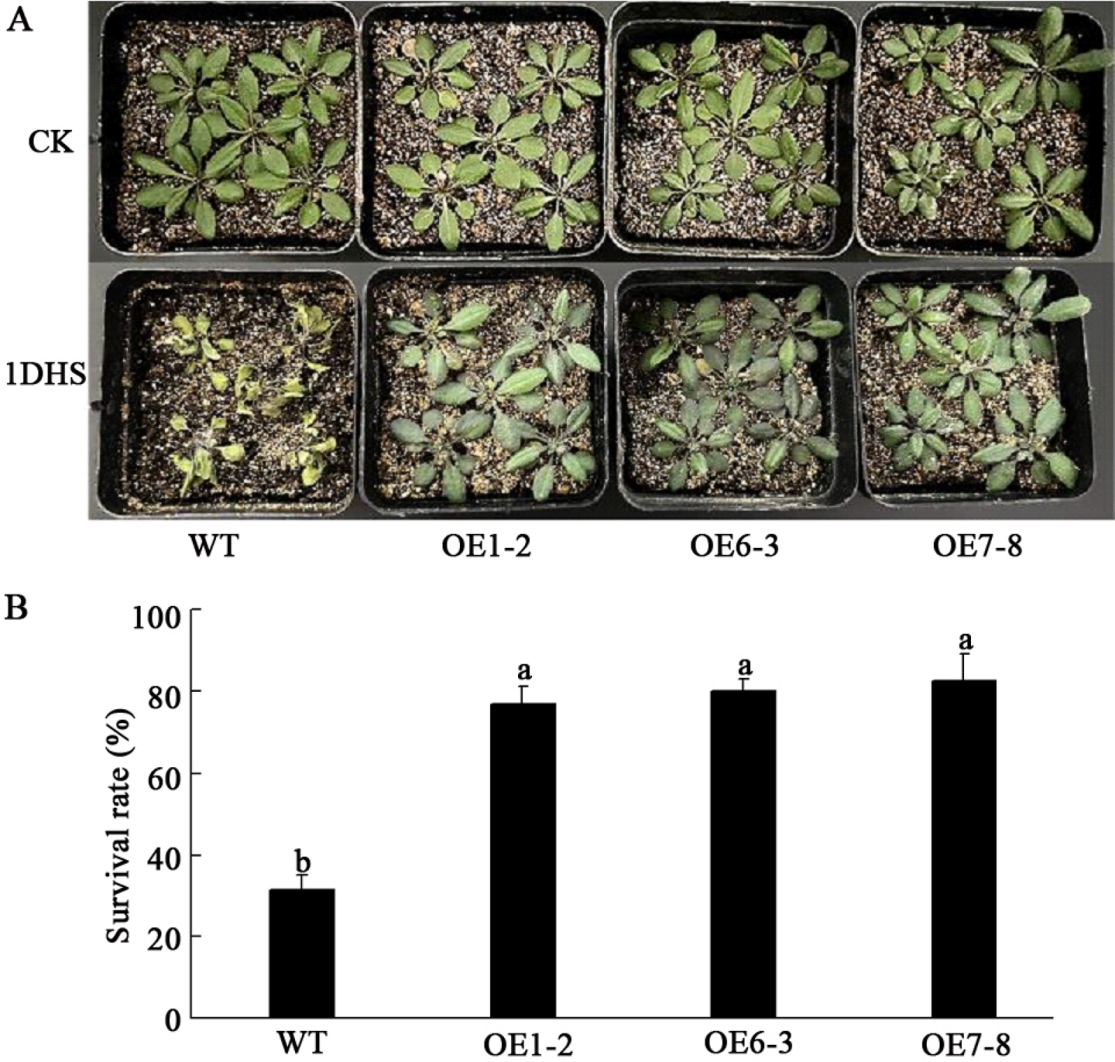
Heat tolerance of WT and transgenic *Arabidopsis* plants. Growth observation (**A**) and survival rate statistics (**B**) of WT and transgenic *Arabidopsis* under 42 °C heat stress for 1 day. Different letters indicate significant differences at *P* < 0.05. 1DHS represents 1 d of heat stress.

The heat tolerance of the transgenic and WT *Arabidopsis* plants was further evaluated by exposure to heat stress. After 1DHS, the survival rate of the WT line plants was only 31.8%, whereas the survival rates of the OE1-2, OE6-3, and OE7-8 lines were 76.9%, 80.3%, and 81.9%, respectively (Fig. 5). These results indicate that the overexpression of *PtWRKY2* in *Arabidopsis* could significantly enhance heat tolerance.

### Overexpression of *PtWRKY2* improved the ROS scavenging capacity of *Arabidopsis* under high temperature conditions

**Figure 6:**
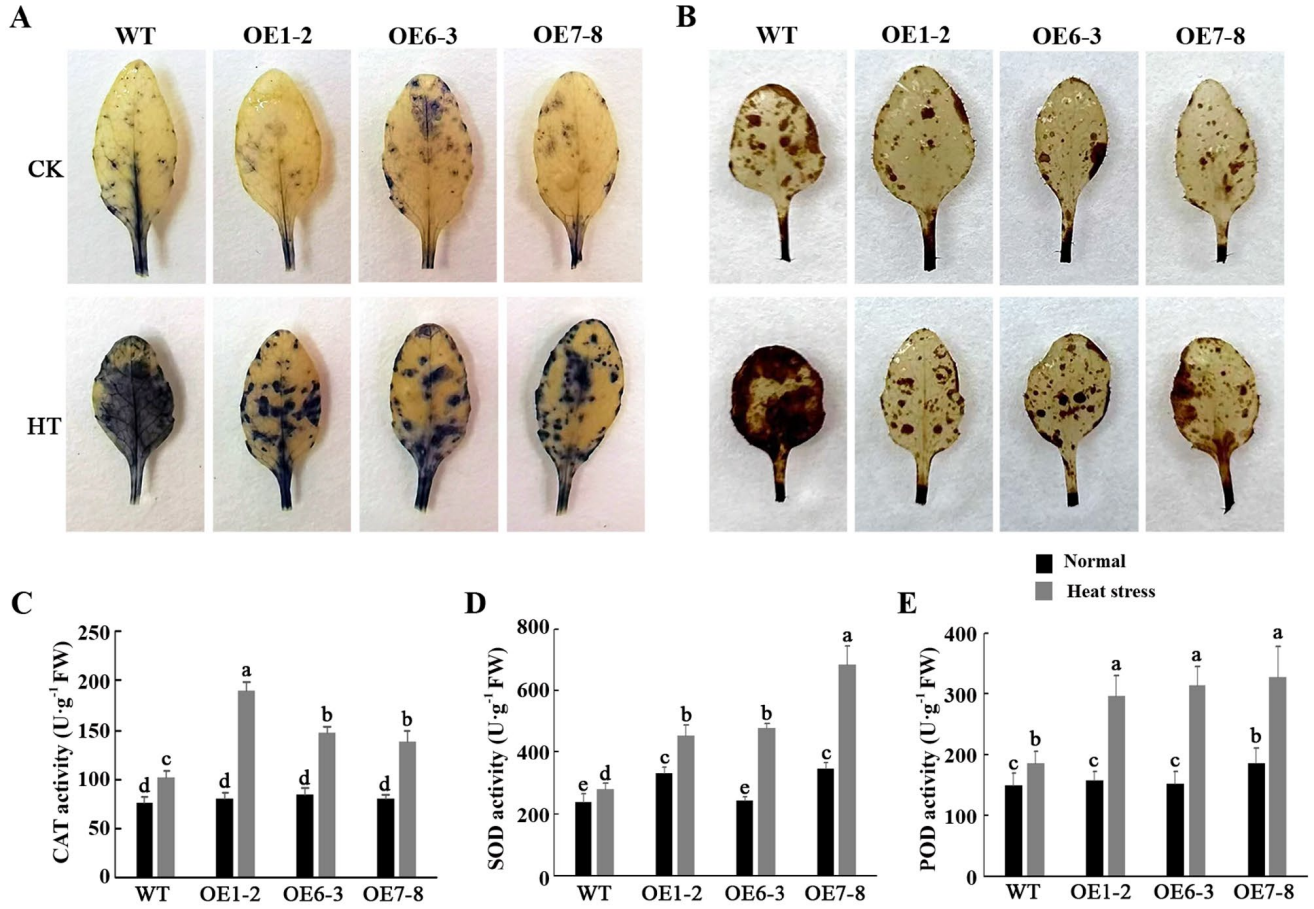
Detection of reactive oxygen species (ROS) accumulation and antioxidant enzyme activity in WT and transgenic *Arabidopsis* plants under heat stress. Leaves of 3-week-old *Arabidopsis* plants grown under normal or heat stress conditions were sampled for (**A**) DAB and (**B**) nitro blue tetrazolium (NBT) staining and (**C**–**E**) catalase (CAT), superoxide dismutase (SOD), and peroxidase (POD) activities. Different letters indicate significant differences at *P* < 0.05.

It is well known that plant senescence induced by abiotic stress is usually associated with the accumulation of reactive oxygen species (ROS), particularly H_2_O_2_ and O_2_^-^. The levels of H_2_O_2_ and O_2_^-^ in the leaves were detected by DAB and NBT staining. The *PtWRKY2*-overexpressing lines exhibited lighter histochemical staining than the WT leaves under heat stress conditions, whereas no significant difference was observed under control conditions (Fig. 6A,B). The activities of PtCAT, PtSOD, and PtPOD were determined in transgenic and WT *Arabidopsis*. The results revealed that the PtCAT, PtSOD, and PtPOD activities were similar in the WT and transgenic *Arabidopsis* under normal conditions. When exposed to high temperatures, the activities increased in both WT and transgenic lines; however, the increment in transgenic plants was higher than that in WT plants (Fig. 6). These results suggest that PtWRKY2 induces the activities of PtCAT, PtSOD, and PtPOD, thereby reducing ROS accumulation in transgenic *Arabidopsis*.

### Overexpression of *PtWRKY2* altered the transcriptional expression levels of heat shock associated genes

**Figure 7:**
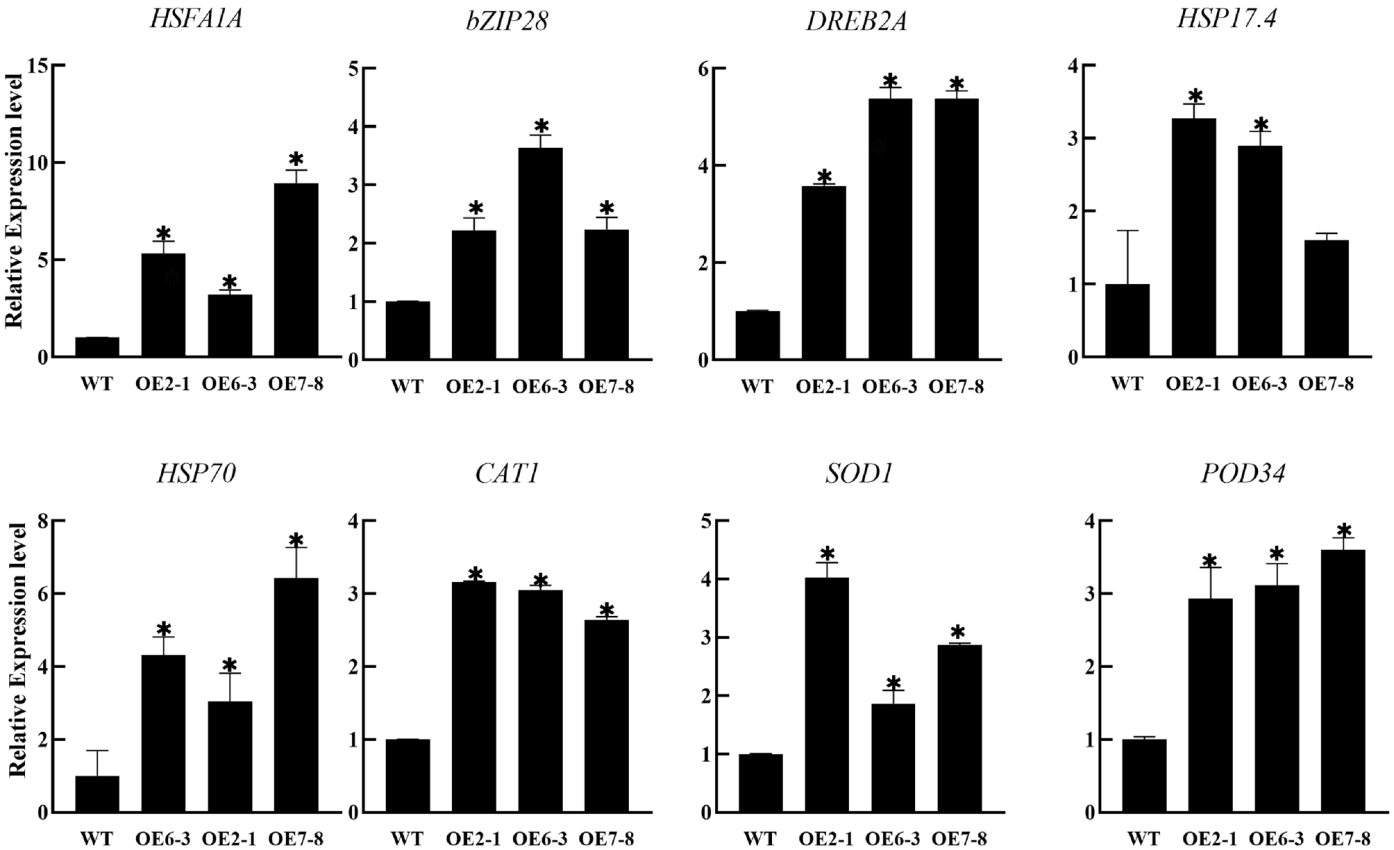
Expression of heat shock-associated genes in WT and transgenic *Arabidopsis* plants under heat stress. * indicates significant differences compared to WT (*P* < 0.05).

Because the overexpression of *PtWRKY2* enhanced the high-temperature tolerance of *Arabidopsis*, the expression profiles of heat shock-associated genes were analyzed in the WT and transgenic lines under high-temperature conditions. The expression levels of five heat shock-related transcription factor-encoding genes, *HSFA1A*, *HSFA7A*, *bZIP28*, *DREB2A,* and *DREB2B,* were significantly higher in the *PtWRKY2* overexpressed lines compared to that in the WT plants. Moreover, three genes related to antioxidant protection (*POD34*, *CAT1,* and *SOD1*) were upregulated in the transgenic lines. In addition, the overexpression of *PtWRKY2* increased the transcript levels of the heat shock protein-coding genes *HSP70* and *HSP17.4* (Fig. 7). These results suggest that PtWRKY2 enhances the expression of heat shock-associated genes, thereby conferring heat tolerance to plants.

## Discussion

WRKY transcription factors are widely distributed in plants and have been identified in many species, including *Arabidopsis*^23^, rice^24^, and wheat^25^, etc. WRKY proteins play an important regulatory role in many life processes and stress responses, and act as an important part of the plant stress signal transduction pathway^26–28^. In the present study, we identified five *PtWRKY* genes and analyzed the function of *PtWRKY2* in response to heat stress, which could be helpful in understanding the transcriptional regulation of ST in *P. ternata* under high-temperature stress.

*WRKY* expression is usually tissue specific and is often affected by stress, thereby playing a role in regulating various biological processes in plants. In peppers, *CaWRKY40* is induced by heat stress and functions as a positive regulator of heat stress tolerance^29^. In potatoes, the leaf-specific genes *StWRKY016*, *StWRKY045,* and *StWRKY055* act as regulators of heat stress^30^. In accordance with these reports, *PtWRKY2* was highly expressed in the leaves of *P. ternata* and significantly upregulated at high temperatures. Furthermore, PtWRKY2 is closely related to AtWRKY30, which enhances thermotolerance in wheat^31^, which suggests that *PtWRKY2* most likely plays an important role for *P. ternata* in terms of the response to heat stress. In addition to heat stress-related *WRKY* genes in *Arabidopsis*^13^, *WRKYs* have been shown to function in high temperature tolerance in other species. For example, overexpression of *OsWRKY11* increases the heat resistance of rice, whereas *TaWRKY1* and *TaWRKY33* confer heat tolerance in *Arabidopsis* ^32,33^. Here, the overexpression of *PtWRKY2* in *Arabidopsis* increased seed germination, root elongation, and seedling survival at high temperatures, suggesting that *PtWRKY2* positively regulates thermotolerance. Basing on the phylogenetic analysis, PtWRKY2 is also grouped together with AtWRKY53 and OsWRKY72, which is related with the drought stress signal^34,35^. Thereby, it is speculated that PtWRKY2 probably also functions in the drought stress of *P. ternata.*

Heat stress often leads to the excessive accumulation of ROS in plants, causing oxidative damage and leading to plant senescence^36^. In *P. ternata*, ROS accumulation and antioxidant enzyme activity fluctuate under heat stress^37,38^, suggesting that dynamic ROS accumulation is closely related to senescence. Previous studies have revealed that some WRKYs participate in the ROS clearance pathway; for example, transgenic rice plants overexpressing *OsWRKY2* displayed increased ROS accumulation, whereas *AtWRKY57* could confer ROS clearance^39,40^. Here, we found that overexpression of *PtWRKY2* contributes to ROS elimination in *Arabidopsis*, which suggests that PtWRKY2 enhances thermotolerance, possibly by regulating ROS clearance.

When plants encounter heat stress, a transcriptional network is activated to regulate the expression of thermoresponsive genes^41^. It has reported that HSF1s usually play a major role in these signaling networks^42^. In addition, *DREB2A* and *bZIP28* are important heat shock response-related genes; mutant plants are usually hypersensitive to heat stress^43,44^. The upregulation of these genes (*HSFA1A*, *DREB2A,* and *bZIP28*) in *PtWRKY2* transgenic plants suggested that *PtWRKY2* may act as an important regulator in heat shock signal networks. Heat shock proteins (HSPs) are induced by high temperatures, and act as molecular chaperones that enhance thermotolerance^45^. A previous study revealed that WRKY family members could induce the expression of certain *HSPs*^46^, and we found *HSP70* and *HSP17.4* were both induced in the *PtWRKY2* overexpressing lines, further suggesting that *PtWRKY2* enhances thermotolerance, most likely by increasing the transcription of certain *HSPs*. Coinciding with the high activities of CAT, SOD, and POD, the expression of *CAT1*, *SOD1* and *POD34* was upregulated in the transgenic lines under heat stress, which is in line with the findings of Arabidopsis MEKK1^47^. Collectively, the transcriptional regulatory function of PtWRKY2 in response to heat stress was explored; however, the location of PtWRKY2 in the signaling networks remains unclear and requires further research.

## Materials and methods

### Plant materials and growth conditions

*P. ternata* tubers (1 cm in diameter) were selected from the Experimental Farm of Huaibei Normal University (N 33°16′, E 116°23′, altitude: 340 m) and planted in potting soil. The potted plants were kept in a phytotron with a 16 h photoperiod and 35 µmol m^−2^ s^−1^ light intensity at 25 °C. When *P. ternata* reached the three-leaf stage, with a height of approximately 15 cm, its leaves, petioles, tubers, and roots were collected. To induce high-temperature stress, the three-leaf-stage seedlings were exposed to temperatures of 42 °C, while the photoperiod and light density remained unchanged. Whole plants were collected after 0, 4, 12, and 24 h of high-temperature stress. Each sample consisted of three plants, and three biological replicates were used for each treatment.

Col-0 background *Arabidopsis* was used as the wild-type (WT) line, and all *Arabidopsis* seeds were vernalized for 3 days and sown in a sterilized mixture with three parts nutrient soil and one part vermiculite. The seedlings were exposed to a 16 h light (50 µmol m^−2^ s^−1^) and 8 h dark cycle at 23 °C.

### Identification and bioinformatics analysis of WRKY family proteins in *P. ternata*

The WRKY protein in *P. ternata* was searched in our previous transcriptome data^19^ based on the sequences of WRKY conserved domains in *Arabidopsis*. Candidate WRKY genes of *P. ternata* were obtained by BLASTP analysis using a hidden Markov model of WRKY. The molecular weights (MWs) and isoelectric points (pIs) of the WRKY proteins were predicted using the ProtParam tool (https://web.expasy.org/protparam/). The domains and conserved domains of the PtWRKY proteins were analyzed using Pfam (http://pfam.xfam.org/) and MEME (http://meme-suite.org/index.html). Finally, TBtools software was used to produce the visualization diagram. The WRKY proteins from *Arabidopsis* and *Oryza* were downloaded from the NCBI database^48^, and the phylogenetic tree of WRKY proteins in *P. ternata*, *Arabidopsis,* and *Oryza* was established using MEGA7.0 software and the Maximum Likelihood method, with the bootstrap setting as 1000.

### Subcellular localization assay

The coding sequence of PtWRKY2 was amplified and then transformed into the vector pCAMBIA1302 for constructing a fusion plasmid, named 35S-PtWRKY2-GFP. The resulting 35S-PtWRKY2-GFP plasmid and empty pCAMBIA1302 control were transformed into tobacco epidermal cells refering to a previously published method^22^. GFP signals from the tobacco epidermal cells were captured under a fluorescence microscope (PA53 FS6, Motic, China).

### Transcriptional activation activity assay

The full-length coding sequence of *PtWRKY2* was cloned and fused to the GAL4 DNA-binding domain (DB) in the pGBKT7 vector to generate a recombinant vector, pGBKT7-PtWRKY2. The pGBKT7-53 plasmid and pGBKT7 empty vector were used as positive and negative controls, respectively. All plasmids were transformed into the Y2HGold yeast strain, which were subsequently cultured on SD/-Trp and SD/-Trp/-His/-Ade plates with or without X-α-gal. After 3–5 days’ incubation of the yeast cells at 30 °C, the transactivation activity of *PtWRKY2* was evaluated using a previously reported method^28^.

### Dual-luciferase activity assay

A dual-luciferase activity assay of PtWRKY2 and W-box was performed according to a previously reported method^22^. Briefly, the PtWRKY2 coding sequence was amplified and transformed into the pGreenII62-SK vector to produce a fusion vector, PtWRKY2-62-SK. The three repeats of the W-box (TTGACY) and mW-box (TTTAAY) were synthesized by oligonucleotide sequencing and cloned into the vector pGreenII-0800 to generate W-box-0800 and mW-box-0800, respectively. Using pGreenII-0800 as a control, the plasmid PtWRKY2-62-SK was co-transformed into *N. benthamiana* leaves with W-box-800, mW-box-800, and pGreenII-0800, respectively. After 72 h transfection, LUC signals were captured in the leaves using a multi-chemiluminescent imaging system (Tanon 5200, China). The activity of LUC/REN was determined using the Dual-LUC Assay Kit (Yeasen, China).

### Plasmid construction and acquisition of transgenic plant material

The 1053 bp coding sequence of *PtWRKY2* was amplified using its specific primers (Table S1), and introduced into the multiple cloning sites behind the *CaMV 35S* promoter in the pCAMBIA1301a vector; the result was an overexpression vector of the *PtWRKY2* construct. The recombinant plasmid was transformed into Col-0 *Arabidopsis* via the *Agrobacterium*-mediated floral-dip method^49^. The T_3_ homozygous lines were obtained for subsequent experiments.

### High temperature tolerance analysis of transgenic plants

Newly harvested seeds from the WT and T_3_ pure transgenic lines were selected for germination experiments. The seeds were sown on 1/2 MS solid media after surface sterilization, and treated with 4 °C for 3 days’ vernalization. Thereafter, the seeds were kept at 23 °C for 1 day and subsequently exposed to a temperature of 42 °C for 0, 2, and 3 days. Next, seeds were cultured at 23 °C and captured at 14 days for germination rate calculations. For the root length experiments, the seeds were germinated on 1/2 MS solid media at 23 °C. When the root length reached approximately 0.5 cm, half of the germinated seeds were exposed to temperatures of 42 °C for 1 day, while the others were kept at 23 °C; the root length was measured after 10 days of growth. For the seedling survival rate assay, three-week-old WT and transgenic *Arabidopsis* plants were exposed to temperatures of 42 °C for 24 h, and subsequently transferred to a 23 °C environment for the survival rate calculation. Each experiment was repeated thrice.

### Determination of ROS and antioxidant enzyme activity

Three-week-old WT and transgenic *Arabidopsis* plants were exposed to temperatures of 42 °C for 12 h, and the leaves were collected for reactive oxygen species (ROS) and antioxidant enzyme activity detection. The accumulation of hydrogen peroxide (H_2_O_2_) and superoxide anion (O_2_^−^) in the leaves were analyzed via staining with 3,3′-DAB and nitro blue tetrazolium (NBT), respectively. The catalase (CAT), superoxide dismutase (SOD), and peroxidase (POD) activities were measured using a previously reported method^50^.

### RNA extraction and quantitative real-time PCR

Total RNA was extracted from *P. ternata* or *Arabidopsis* samples using TRIzol reagent (Invitrogen), following a previously reported procedure^18,50^. The RNA was then used as a template for cDNA synthesis using the MonScript™ RT III × All-In-One transcription kit, which was obtained from Monad (Chuzhou, China). Quantitative real-time PCR was performed using SYBR Premix (Roche) on a LightCycler 96 system (Roche, Basel, Switzerland). *Pt18S* and *AtTUB2* were selected as internal references for *P. ternata* and *Arabidopsis*, respectively. Each assay was run with three biological replicates, and the relative expression levels were calculated based on the 2^−△△CT^ method. The primer sequences used in this study are listed in Table S1.

### Statistical analysis

Data were analyzed using SPSS Statistics 22 software and the data presented in the figures represent the means ± SD values of three biological replicates. Statistical significance was assessed using Duncan’s multiple range test or the Student’s t-test, with significance set at* P* < 0.05.

### Ethical statement

The authors declare that all the plant experiments/protocols were performed with relevant institutional, national, and international guidelines and legislation.

## Conclusions

In summary, five *PtWRKYs* were identified from the transcriptome data while *PtWRKY2* was induced and the other four *PtWRKYs* were suppressed under high temperatures. The overexpression of *PtWRKY2* significantly improved the heat tolerance of *Arabidopsis*, inluding seed germination, root growth and survival rate. RT-qPCR revealed that PtWRKY2 could up-regulate heat shock-associated genes, and decreased the ROS accumulation under high temperature, thereby to enhance the heat tolerance in *Arabidopsis*. This study firstly identified the function of *PtWRKY2* and laid a foundation for further exploring the transcriptional regulation of PtWRKYs in the heat response of *P. ternata*.

### Supplementary Information

Supplementary Information.

## Data Availability

Data will be made available from the corresponding author on request.
