# Supplementary material for: PtWRKY2, a WRKY transcription factor from Pinellia ternata confers heat tolerance in Arabidopsis
Source: Sci Rep. 2024 Jun 14;14:13807. doi: 10.1038/s41598-024-64560-0 (PMC11178784; doi:10.1038/s41598-024-64560-0)
Supplement: Supplementary file 1 — Supplementary Information. [file 41598_2024_64560_MOESM1_ESM.doc]

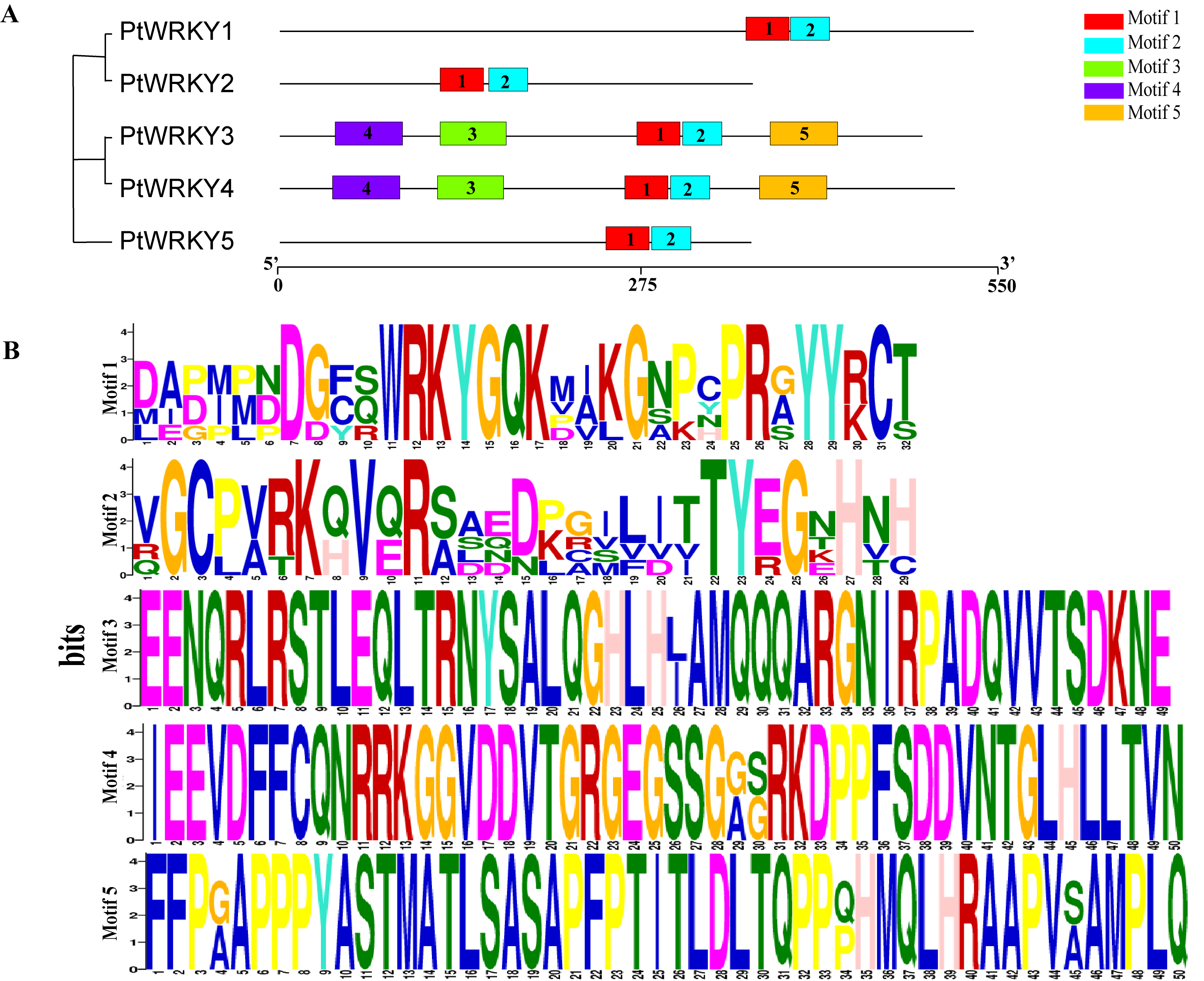


Figure S1 Conserved motifs (A) and conserved sequence (B) of PtWRKY1-5 in *Pinellia ternata*.

Table S1. Primers used in this study

| **Used for** | **Primer Name** | **Sequences (5’-3’)** |
| --- | --- | --- |
| qRT-PCR | *Pt18SrRNA*-F | CGCATATAAATAAACGGAGGAA |
|  | *Pt18SrRNA*-R | GACGCTTCTACAGACTACA |
|  | *PtWRKY1*-F | ATGATGAGGGGAATGTTCG |
|  | *PtWRKY1*-R | AACAGGTACAACTAGCTCTG |
|  | *PtWRKY2*-F | CCTGACGAATGAAAGACTA |
|  | *PtWRKY2*-R | ACTTCTTCTGCATCTCCTTG |
|  | *PtWRKY3*-F | GTTCGAGAGATCTGAACCTT |
|  | *PtWRKY3*-R | CTGAACATTCCCCTCATCAT |
|  | *PtWRKY4*-F | CAAGTTTGGGATGGAGAAA |
|  | *PtWRKY4*-R | AAAGACATATTGGAGGCGT |
|  | *PtWRKY5*-F | AGACCTTCCAACACCAAAC |
|  | *PtWRKY5*-R | GTTCCTGTTGTCTCCATTCT |
|  | *PtWRKY6*-F | TTTGACTACATCACCACCAC |
|  | *PtWRKY6*-R | CTTGATCATCTGCAGGATCT |
|  | *PtWRKY7*-F | CATAAGGAAGAACCCCCAT |
|  | *PtWRKY7*-R | CATCTTGGATATGTTGGGCA |
|  | *AtHSP70*-F | AACATGACCGCGTCGAAATC |
| **Used for** | **Primer Name** | **Sequences (5’-3’)** |
| qRT-PCR | *AtHSP70*-F | AACATGACCGCGTCGAAATC |
|  | *AtHSP70*-R | TCGCTGTCAGTGAAAGCAAC |
|  | *AtBZIP28*-F | TTCGTTGAGCAATGCCAGTG |
|  | *AtBZIP28*-R | TTTCTCGCTCGCCAAAACAG |
|  | *AtSOD1*-F | TGCCACCTTCACAATCACTG |
|  | *AtSOD1*-R | TCTGCATGGACAACAACAGC |
|  | *AtCAT1*-F | ACAGGCAAGAACGATTCGTG |
|  | *AtCAT1*-R | TCCAAATGCTGCGGATTTCG |
|  | *AtDREB2A*-F | AACCTGTCAGCAACAACAGC |
|  | *AtDREB2A*-R | AAGCCTGCAAACACATCGTC |
|  | *AtDREB2B*-F | ATTGGGGCCAAGTTTTGCAG |
|  | *AtDREB2B*-R | AAATCCGGTTGCAGCTGTTG |
|  | *AtHSP17.4*-F | TTTCGGTTGCCAGAGAATGC |
|  | *AtHSP17.4*-R | TGAACTTTCGGCACCGTAAC |
|  | *AtHSFA1A*-F | TCTCAGGGTCAAGGTTCAATGG |
|  | *AtHSFA1A*-R | TTTGTTGCTGCTGGCGTAAC |
|  | *AtHSFA7A*-F | GACAGCAGCAACAAACAACG |
|  | *AtHSFA7A*-R | AACGAAGGACTCTGCATTGC |
|  | *AtPOD34*-F | CAATGCCACTGACACAATCC |
|  | *AtPOD34*-R | GAGCAGAGAGTTGGAGTTCACA |
| Semi-quantitative RT-PCR | *TUB2*-F | GACTGTCTCCAAGGGTTCCA |
|  | *TUB2*-R | GCTTTCGGAGGTCAGAGTTG |
|  | *PtWRKY2*-F | CTGGAGGAAGTACGGGCAGAAGGACA |
|  | *PtWRKY2*-R | GGCGAGATGAACGGCGAGGAGAA |
| Cloning of PtWRKY2 gene | *PtWRKY2*-F | ATGGAGAAGTGCGGCGCC |
|  | *PtWRKY2*-R | TTAGGGTTCCCCCCGAAGC |
| Overexpression vector construction | PtWRKY2-1301a-F | GGGGTACCATGGAGAAGTGCGGCGCC |
|  | PtWRKY2-1301a-R | GCTCTAGATTAGGGTTCCCCCCGAAGC |
| Subcellular localization | PtWRKY2-SL2-F | GTCCGGAGCTAGCTCTAGAATGGAGAAGTGCGGCGCC |
|  | PtWRKY2-SL2-R | CCTTGCTCACCATGGATCCTTAGGGTTCCCCCCGAAGC |
| Transactivation activity assays | PtWRKY2-BK2-F | GGCCATGGAGGCCGAATTCATGGAGAAGTGCGGCGCC |
|  | PtWRKY2-BK2-R | GCTGCAGGTCGACGGATCCTTAGGGTTCCCCCCGAAGC |
| Dual-luciferase reporter assaysPtWRKY2-62-SK-F | CGGTGGCGGCCGCTCTAGAATGGAGAAGTGCGGCGCC |  |
|  | PtWRKY2-62-SK-R | CGACGGTATCGATAAGCTTTTAGGGTTCCCCCCGAAGC |
|  | WRKYRS-0800-F | CGTTGACYGTTGACYGAGTTGACYTTTTTA |
|  | WRKYRS-0800-R | AGCTTTAAAAARGTCAACTCRGTCAACRGTCAACGGGTAC |
|  | mWRKYRS-0800-F | CCGTTAAAYGTTAAAYGAGTTAAAYTTTTTAA |
|  | mWRKYRS-0800-R | AGCTTTAAAAARTTTAACTCRTTTAACRTTTAACGGGTAC |

Table S2 The physicochemical properties of five *WRKY* transcription factors in *P. ternata*

| Transcription factor | Amino acid/number | Relative molecular weight/kD | Theoretical isoelectric point |
| --- | --- | --- | --- |
| PtWRKY1 | 515 | 55.07 | 9.23 |
| PtWRKY2 | 350 | 38.19 | 10.15 |
| PtWRKY3 | 477 | 50.82 | 8.12 |
| PtWRKY4 | 501 | 53.20 | 7.67 |
| PtWRKY5 | 351 | 37.791 | 6.1 |
